# Maternal depressive symptoms and early childhood development: the role of mother–child interactions among mother–child dyads in rural areas of Central and Western China

**DOI:** 10.7717/peerj.11060

**Published:** 2021-03-30

**Authors:** Xiaoli Liu, Chenlu Yang, Yuning Yang, Xiaona Huang, Yinping Wang, Yaqing Gao, Qiying Song, Yan Wang, Hong Zhou

**Affiliations:** 1 Research Center of Clinical Epidemiology, Peking University Third Hospital, Beijing, China; 2 Department of Nutrition and Food Hygiene, School of Public Health, Peking University, Beijing, China; 3 United Nations International Children’s Emergency Fund China, Beijing, China; 4 Department of Maternal and Child Health, School of Public Health, Peking University, Beijing, China

**Keywords:** Maternal depressive symptoms, Early childhood development, Mother–child interactions, Mediation analyses, Moderation analyses

## Abstract

**Background:**

The associations among maternal depressive symptoms (MDS), mother–child interactions and early child development are poorly understood. This study aimed to explore the role of mother–child interactions on the associations between MDS and child development.

**Methods:**

A cross-sectional study with a multistage sampling method was conducted in rural areas of Central and Western China. MDS, child development outcomes (communication, gross motor function, fine motor function, problem solving and personal social skills) and mother–child interactions were assessed by The Edinburgh Postpartum Depression Scale, the Chinese version of the Ages and Stages Questionnaires and the Multiple Indicator Cluster Surveys, respectively. Regression-based statistical mediation and moderation were conducted using the PROCESS macro for SPSS.

**Results:**

A total of 2,548 participants (mothers: 1,274; children: 1,274) were included in our analyses. MDS was negatively associated with child development outcomes and mother–child interactions partly mediated these associations. The proportion of the mediating effect of mother–child interactions was 7.7% for communication, 8.2% for gross motor, 10.3% for fine motor, 10.1% for problem-solving and 9.5% for personal social domains. In addition, the interaction effects of MDS and mother–child interactions on the communication domain were significant (*β* = 0.070, 95% CI 0.016, 0.124; *p* = 0.011). The associations between MDS and child communication abilities were weaker at the high level (simple slope = −0.019, *t* =  − 0.458, *p* = 0.647) of mother–child interactions than at the mean level (simple slope = −0.089, *t* =  − 3.190, *p* = 0.002) and the low level (simple slope = −0.158, *t* =  − 4.231, *p* < 0.001). Similar moderating effects were not observed in the other child development outcomes.

**Conclusion:**

Our results suggest the important role of mother–child interactions on the associations between MDS and early childhood development. Due to the cross-sectional design of this study, these associations require further investigation in prospective studies.

## Introduction

The early years of life are a critical period for child brain growth and development, with the potential to later impact social, economic and health-related quality of life (Peacock-Chambers, Ivy & Bair-Merritt, 2017). One research study indicates that 250 million children (43%) younger than 5 years in low-income and middle-income countries are at risk of not reaching their developmental potential (Black et al., 2017). A large number of studies have confirmed maternal depressive symptoms (MDS) as a predictor of various negative child development outcomes, such as poor physical and cognitive development, increased behavioural problems and later common mental disorders (Aoyagi et al., 2019; Gelaye et al., 2016; Liu et al., 2017; Netsi et al., 2018; Tuovinen et al., 2018). However, most of these studies have been performed in high-income countries and have samples consisting of participants only representing Western, educated, industrialized, rich, and democratic societies (Rogers et al., 2020). Studies with more diverse samples are needed. Also, in low-to-middle income countries, MDS is more highly prevalent than in high-income countries (Gelaye et al., 2016), and the negative factors that impact both MDS and early childhood development are also more common than in high-income countries (Herba et al., 2016).

Understanding the pathways that link MDS and child development outcomes is a critical step in early intervention. MDS has considerable impact on mother–child interactions because depressed mothers tend to fail to bond well with their babies (O’Higgins et al., 2013). In addition, depressed mothers have increased self-focus and psychological distancing as well as decreased mother–child involvement, warmth, sensitivity and contingent responsiveness (Hakanen et al., 2019; Humphreys et al., 2018; Ierardi et al., 2019; Lanzi, Bert & Jacobs, 2009). A lack of mother–child interactions is associated with high rates of developmental delays in children (Yang et al., 2019; Yue et al., 2019). Taken together, mother–child interactions may play a mediating role in the association between MDS and child development. That is to say, MDS affects mother–child interactions and thereby child development. Furthermore, the interactive effects of MDS and mother–child interactions is also a point of concern. MDS and child development vary as a function of other aspects of the caregiving environment, and the negative or positive parenting behaviours may serve as environmental contexts that exacerbate or attenuate the influence of parental depression on children (Brett et al., 2020; Olino et al., 2016). Therefore, mother–child interactions may play a moderating role in the association between MDS and child development, which means that different levels of mother–child interactions may ameliorate or worsen the negative impact of MDS. However, there has been little discussion about both mediating and moderating effects of mother–child interactions in the association between MDS and child development.

Over the past few decades, there has been a tremendous amount of economic development in China. However, the economic development has not been uniform throughout the country, and the resulting regional economic development gaps are still of great concern to society because there are increased health risks in less developed regions. A meta-analysis reported that the prevalence of maternal postpartum depression in China is lower in the more developed Eastern China compared with the less developed Central China and Western China (Qian & Yan, 2013). Furthermore, children in more economically disadvantaged regions, such as rural areas, may be more affected by problems associated with MDS (Meaney, 2018). Therefore, MDS and the associated negative impacts may be more prevalent among mother–child dyads in rural areas of Central and Western China. However, although some studies have reported the negative impacts of MDS on child development in China (Ip et al., 2018; Li et al., 2018; Lin et al., 2017; Wang et al., 2018; Wei et al., 2018; Yue et al., 2018), most of these studies have been conducted in urban areas. The similar studies in rural China start quite late. In addition, only a few of them have taken mother–child interactions into consideration at the same time.

**Figure 1:**
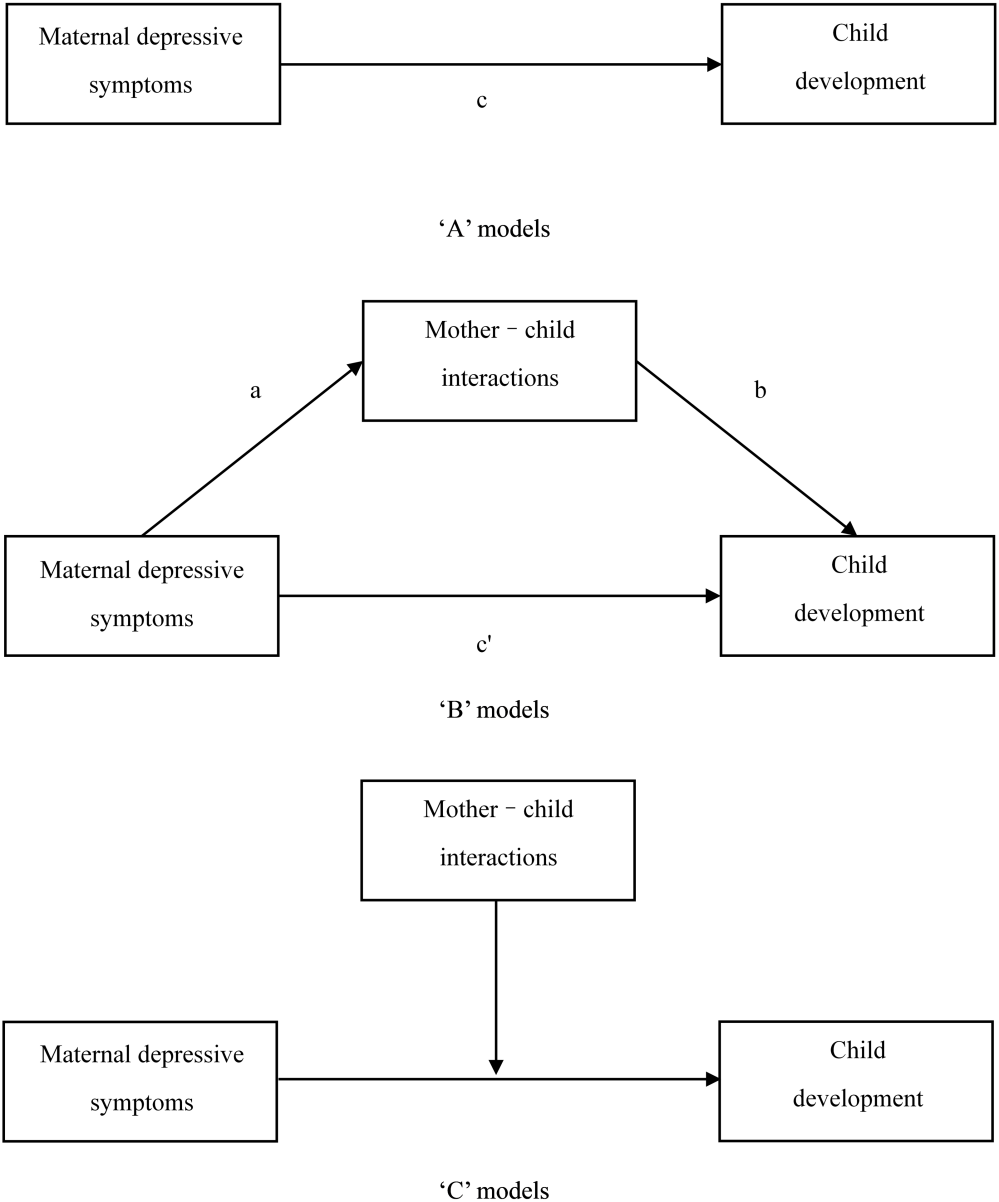
The hypothesis models in this study.

Therefore, the objective of the current study was to examine the associations among MDS, mother–child interactions and child development in rural areas of Central and Western China. We hypothesised that MDS impacts child development and that mother–child interactions function as mediators and/or moderators of the associations between MDS and child development. The hypothesis models are illustrated in Fig. 1.

## Materials & Methods

### Participants

This study was based on a subset of data from a cross-sectional survey in eight rural counties of four provinces in Central and Western China in the year of 2016 to 2017, namely Xinjiang, Qinghai, Jiangxi and Ningxia. Research teams from the United Nations International Children’s Emergency Fund (UNICEF), Peking University, Lanzhou University and Capital Medical University as well as government officials and field staff worked together on this survey. In China, there are many administrative villages in each county, and each administrative village can be composed of one or more natural villages. Natural villages are naturally formed and have natural boundaries. A three-stage cluster sampling method was applied in each county. First, 15 administrative villages per county were selected and then two natural villages per administrative village were selected at random with population proportional to size (PPS) (WHO, 2015). Third, within each selected natural village, a simple random sampling method was used to select eight families that had at least one child under five years. Inclusion and exclusion criteria for the study were provided in detail elsewhere (Yang et al., 2019). Mothers that were the primary caregivers were evaluated for depressive symptoms.

### Measures

#### MDS

The Edinburgh Postpartum Depression Scale (EPDS) was used to identify MDS. EPDS has been validated in Chinese mothers (Lau et al., 2010). The EPDS questionnaire has 10 items that each have four possible answers. For each question, the mother was instructed to choose one of four possible answers that best described what she had experienced in the previous seven days. Each answer was converted to a score on a scale of 0 to 3. The total score ranged from 0 to 30 with higher scores representing a higher severity of depressive symptoms.

#### Mother–child interactions

UNICEF’s Multiple Indicator Cluster Surveys (MICS) questionnaire was used to assess mother–child interactions based on the activities that a mother engaged in with her child in the last three days (Bornstein & Putnick, 2012; UNICEF, 2013; Zhang et al., 2018a). The total number of these behaviours engaged in by the mother was equivalent to the score that was given. For example, if the mother engaged in one type of behaviour, the score would be 1. The total score ranged from 0 to 6. A higher score represents more mother–child interactions.

#### Child development outcomes

The Chinese version of the Ages & Stages Questionnaires, Third Edition (ASQ-C), was used to evaluate child development outcomes in our study. The ASQ-C is a series of parent–completed questionnaires designed to screen and monitor the development of children aged 1–66 months, which has been found to be a reliable and valid screening instrument for development outcomes in Chinese children (Squires & Bricker, 2009; Wei et al., 2015). The sensitivity of the ASQ-C was 87.50% and the specificity of the ASQ-C was 84.48% (Wei et al., 2015). The ASQ-C consists of 21 questionnaires with each questionnaire for a different age range of children. The ASQ-C contains five major developmental domains (communication, gross motor function, fine motor function, problem solving and personal social skills) and each domain consists of six questions. The mother was asked to answer questions about some things that her child could and could not do. For each question, an answer of ‘yes’ was scored 10 points, an answer of ‘sometimes’ was scored 5 points and an answer of ‘not yet’ was scored 0 points. The score on each of the six questions was summed to obtain a domain score, with lower scores representing lower levels of development outcomes.

### Covariates

#### Child malnutrition

Each child’s length/height and weight were measured. Children’s malnutrition status was defined based on their anthropometry along three dimensions: weight-for-age, height-for-age, and weight-for-height (WHO, 2006).

#### Other covariates

Basic characteristics of the children were collected, including sex (male or female), ethnicity (Han and minorities), age (1–11, 12–23, 24–35, 36–47 or 48–59 months), preterm (yes or no), birth order (1 or 2 or more), maternal education (primary school or below, middle school or high school, above high school) and family economic status (poorest, poor, middle, richer and richest). Family economic status was categorised based on the quintiles in the distribution of annual net income. The annual net income was equal to the total family income for the last year minus the total production and operating expenses (Liu et al., 2018; Yang et al., 2019).

#### Statistical analyses

The statistical analyses were conducted in SPSS v.25.0 with the addition of the PROCESS plug-in for mediation and moderation analyses. First, a Pearson’s correlation was used to evaluate the bivariate associations among MDS, mother–child interactions and child development outcomes, and all of these variables were conducted as continuous variables. Second, multiple linear regression analyses were used to determine the associations between MDS and child development outcomes (‘A’ models in Fig. 1). The letter *c* represents path coefficients. The ASQ-C score was the dependent variable, the EPDS score was the independent variable and other basic characteristics were covariates in the multiple linear regression analyses. Third, the mediating and moderating analyses were conducted in PROCESS using least squares regression, and the mother–child interactions score was entered as mediator (‘B’ models in Fig. 1, the letters *a*, *b* and *c’* represent path coefficients) and moderator (‘C’ models in Fig. 1). The variables were standardised before analyses. The significance of mediating and moderating effects of mother–child interactions was tested by the bootstrap method. The 95% confidence intervals (CI) were estimated by the bias-corrected bootstrapping procedure with the number of iterations set to 5,000. If the 95% CI of the indirect effects and interaction terms did not include zero, the mother–child interactions significantly mediated and moderated the associations (Hayes, 2013).

### Human ethics

The Medical Ethics Research Board of Peking University Health Science Centre approved this study (IRB00001052-16041). All participating mothers provided consent via written permission or fingerprint for both their own and their children’s involvement.

## Results

### Basic characteristics

A total of 2,548 participants (mothers: 1,274; children: 1,274) were included in our analyses. The basic characteristics are shown in Table 1. Most of the children were male (52.3%) and 62.2% of the children were ethnic minorities. Nearly half of the children were aged 1–23 months. The percentage of preterm births was 4.8%. In addition, about a third of the children were the first child of their parents. Our analysis found that 5.7% of the children were considered malnourished. Nearly half of the mothers in this study had a middle school education level.

**Table 1:** Basic characteristics of the participants this study.

Characteristics		n	%
Sex of child	Male	666	52.3
	Female	608	47.7
Ethnicity	Han	481	37.8
	Minorities	793	62.2
Age (months)	1–11	306	24.0
	12–23	338	26.5
	24–35	262	20.6
	36–47	209	16.4
	48–59	159	12.5
Preterm^a^	No	1,182	95.2
	Yes	60	4.8
Birth order of child	1	438	34.4
	2 or more	836	65.6
Malnutrition^b^	No	1,184	94.3
	Yes	72	5.7
Maternal education	Primary school or below	314	24.6
	Middle school	667	52.4
	High school or above	293	23.9
Family economic status	Poorest	268	21.0
	Poor	284	22.3
	Middle	212	16.6
	Richer	303	23.8
	Richest	207	16.2

**Notes.**

a 32 missing.

b 18 missing.

### Correlations among the target variables

Table 2 presents the bivariate correlations. We found that MDS was negatively correlated to mother–child interactions as well as positive child development outcomes in the five developmental domains (all *p* <0.05). Conversely, mother–child interactions were positively correlated to positive child development outcomes in the five developmental domains (all *p* <0.01).

**Table 2:** Bivariate correlation among target variables.

	1	2	3	4	5	6	7
1. MDS	1						
2. mother–child interactions	−0.066^*^	1					
3. communication	−0.076^**^	0.154^**^	1				
4. gross motor function	−0.108^**^	0.163^**^	0.383^**^	1			
5. fine motor function	−0.118^**^	0.155^**^	0.370^**^	0.363^**^	1		
6. problem solving	−0.110^**^	0.164^**^	0.369^**^	0.379^**^	0.505^**^	1	
7. personal social	−0.087^**^	0.171^**^	0.453^**^	0.418^**^	0.380^**^	0.435^**^	1

**Notes.**

* *p* < 0.05.

** *p* < 0.01.

### MDS and child development outcomes

The associations between MDS and child development outcomes are shown in Table 3. After adjusting for confounding covariates, the scores of MDS had significant negative associations with the scores of communication (*β* = −0.102), gross motor function (*β* = −0.109), fine motor function (*β* = −0.095), problem solving (*β* = −0.098) and personal social domains (*β* = −0.090) (*p* <  0.01).

**Table 3:** Mediating role of mother–child interactions on the associations between maternal depressive symptoms and child development outcomes in five models.

Outcomes	Path coefficients				indirect effect (*a*∗*b*)	95% CI (*a*∗*b*)
	*c*	*a*	*b*	*c*′		
communication	−0.102^***^	−0.074^*^	0.107^***^	−0.094^**^	−0.008	−0.018, −0.002
gross motor function	−0.109^***^	−0.074^*^	0.121^***^	−0.100^***^	−0.009	−0.019, −0.003
fine motor function	−0.095^**^	−0.074^*^	0.132^***^	−0.085^**^	−0.010	−0.021, −0.003
problem solving	−0.098^***^	−0.074^*^	0.134^***^	−0.088^**^	−0.010	−0.021, −0.003
personal social	−0.090^**^	−0.074^*^	0.116^***^	−0.082^**^	−0.009	−0.019, −0.002

**Notes.**

Controlling for sex of child, ethnicity, age, preterm, birth order of child, malnutrition, maternal education and family economic status. The letter *c* represented path coefficients of associations between maternal depressive symptoms and child development outcomes without any mediator; the letter *a* represented path coefficients of associations between maternal depressive symptoms and mother–child interactions in mediating models; the letter *b* represented path coefficients of associations between mother–child interactions and child development outcomes in mediating models; the letter *c*′ represented path coefficients of associations between maternal depressive symptoms and child development outcomes in mediating models.

* *p* < 0.05.

** *p* < 0.01.

*** *p* < 0.001.

### Mediation analyses

In the mediating models (Table 3), the score of MDS was entered as the independent variable and the score of mother-child interactions was entered as the mediator. After adjusting for confounding covariates, the mediation analysis found that MDS was negatively associated with mother–child interactions (*p* <  0.05). However, mother–child interactions were positively associated with child development outcomes in the five developmental domains (*p* <  0.001). Through the bootstrap method, the 95% CI of indirect effects in the mediating models did not contain zero, which indicates significant mediation effects of mother–child interactions on the associations between MDS and child development outcomes. In addition, the associations between MDS and child development outcomes were still significant, which indicated the partially mediating roles of mother–child interactions in these associations (*p* <  0.01). Coefficients (*a* *b/*c*) were used to assess the proportion of mediation. The proportion of the mediating effect of mother-child interactions was 7.7% for communication, 8.2% for gross motor, 10.3% for fine motor, 10.1% for problem solving and 9.5% for personal social domains.

### Moderation analyses

**Figure 2:**
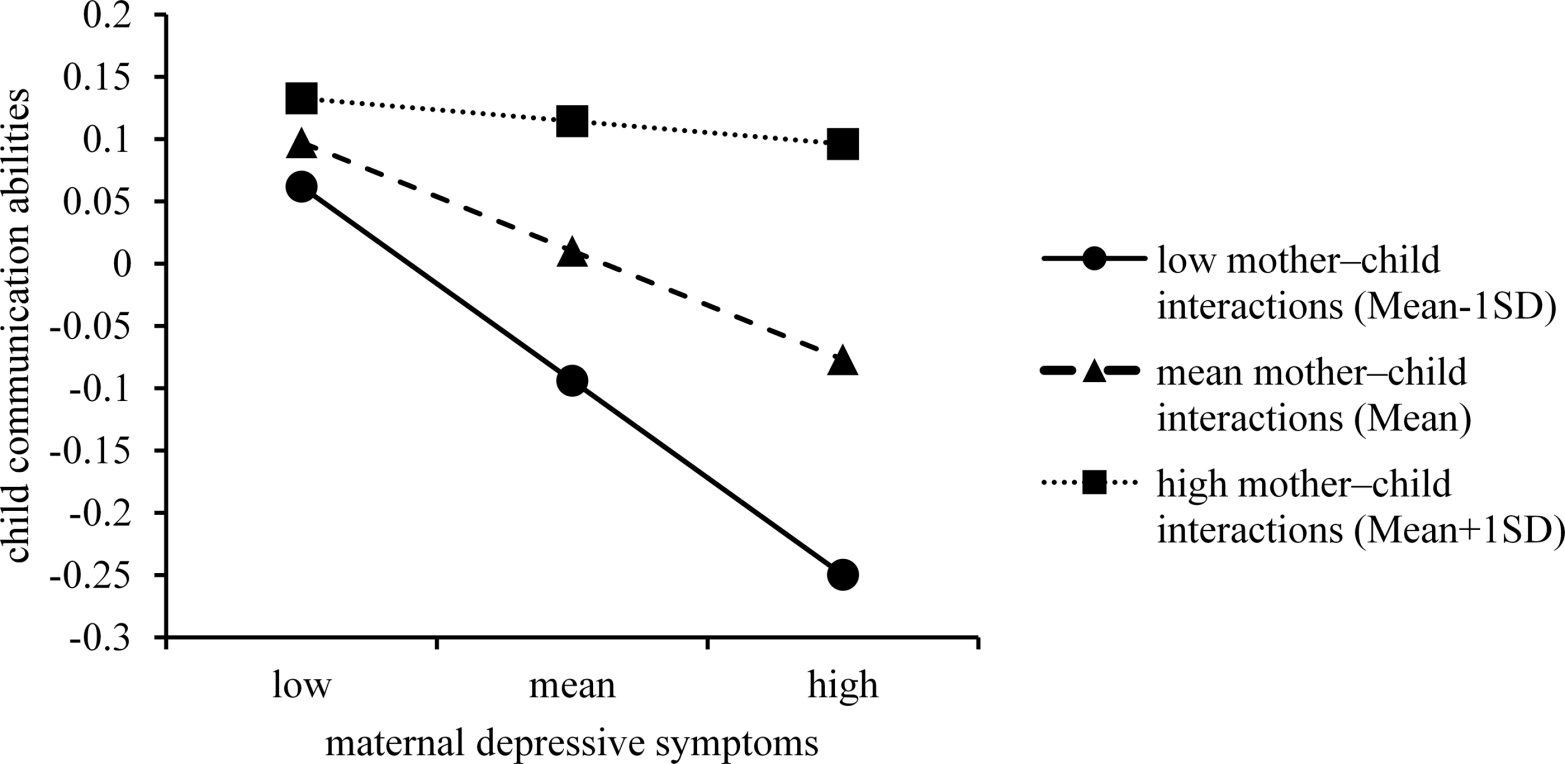
Simple regression lines of maternal depressive symptoms on child communication abilities under different levels of mother–child interactions.

In the moderating models (Table 4), the score of MDS was entered as the independent variable and the score of mother–child interactions was entered as the moderator. After adjusting for confounding covariates, the interaction effects of MDS and mother–child interactions on the communication domain was significant (*β* = 0.070, 95% CI 0.016, 0.124; *p* = 0.011). As depicted in Fig. 2, mother–child interactions were divided into low (the mean minus one standard deviation [SD]), mean (the mean) and high (the mean plus one SD). The associations between MDS and child communication abilities were weaker at the high level (simple slope = −0.019, *t* = −0.458, *p* = 0.647) of mother–child interactions than at the mean level (simple slope = −0.089, *t* =  − 3.190, *p* = 0.002) and the low level (simple slope = −0.158, *t* =  − 4.231, *p* <  0.001).

**Table 4:** Moderating role of mother–child interactions on the associations between maternal depressive symptoms and child development outcomes in five models.

Independent variables	Outcomes	*β*	SE	*p*	95% CI
Maternal depressive symptoms*mother–child interactions	communication	0.070	0.028	0.011	0.016, 0.124
	gross motor function	−0.009	0.028	0.734	−0.064, 0.045
	fine motor function	0.025	0.029	0.395	−0.032, 0.081
	problem solving	−0.004	0.029	0.877	−0.061, 0.052
	personal social	0.012	0.028	0.660	−0.042, 0.067

**Notes.**

Controlling for sex of child, ethnicity, age, preterm, birth order of child, malnutrition, maternal education and family economic status.

## Discussion

Our results revealed the negative impact of MDS on child development outcomes in rural areas of Central and Western China. Furthermore, mother–child interactions partly mediated the associations of MDS with child development in communication, gross motor function, fine motor function, problem solving and personal social skills. In addition, mother–child interactions moderated the associations of MDS with child development in communication.

The wellbeing of the children is tied to the wellbeing of their mothers and the home environment. Consistent with prior research (Yue et al., 2018; Zhang et al., 2018a), our models found negative impacts of MDS on child development, which suggests that future intervention programmes should focus on the depressed mother and her child. The pathways between MDS and child development have received considerable attention because it is critical that interventions are appropriate (Kuckertz, Mitchell & Wiggins, 2018; Wang & Dix, 2015). One potential characteristic of depressed mothers that may be responsible for the negative associations with child development outcomes is disinterest or a lack of engagement. Depressed mothers are less likely to engage in face-to-face play behaviour and interactions with the child, such as vocalising, smiling, imitation and game playing. In addition, depressed mothers are more likely to be more disengaged, irritable and hostile to their children compared to non-depressed mothers (Field, 2010; Ierardi et al., 2019a; Yue et al., 2018; Zhang et al., 2018b). In the current study, we also found that higher MDS was a predictor of decreased mother–child interactions. Furthermore, the absence of mother–child interactions is significantly correlated with child developmental delays in many domains, which is similar to the results of the current study (Weisleder et al., 2018; Yue et al., 2019). We used mediation analyses and found evidence supporting the mediating role of mother–child interactions between MDS and child development in rural China, which is consistent with prior research in other countries (Murray et al., 2010; Stein et al., 2008). Our findings suggest that MDS can indirectly affect child development by influencing mother–child interactions. Thus, identifying and implementing effective assessments and interventions targeted at improving mother–child interactions could help reduce the risk of poor child development caused by MDS.

Previous research has shown that the influence of MDS on child development is moderated by some factors, such as level of financial support, socioeconomic status, suboptimum maternal care, family disharmony and negative emotions of the child (Sacchi et al., 2018; Stein et al., 2014). Whether mother–child interactions could moderate the association between MDS and child development has not previously been examined. Here, we observed a moderating role of mother–child interactions in the child communication domain. In our analysis, a lower level of mother–child interactions strengthened the associations between MDS and lower child communication abilities. Maternal behaviours, such as speech and affect, can be modelled by or transmitted to the child, and the child of a depressed mother may look ‘depressed’ (e.g., less talking, less touching and more negative facial expressions) (Stein et al., 2014; Tronick & Reck, 2009). Importantly, a child’s communication efforts may be ignored by a depressed mother (Valla et al., 2016), and decreased mother–child interactions means that the child loses opportunities to practice and improve communication abilities. Therefore, children exposed to MDS and lower mother–child interactions have a greater chance of poor performance in communication domains.

Despite the moderating effect of mother–child interaction on the communication abilities of the child, we did not observe similar moderating effects in other developmental domains. Therefore, mother–child interactions are more likely to play a mediating role rather than a moderating role on the negative influences of MDS on child development outcomes involving gross motor function, fine motor function, problem solving and personal social abilities. However, mother–child interactions play both mediating and moderating roles on the negative influences of MDS on the communication abilities of the child. A possible explanation for mother–child interactions having both these mediating and moderating effects on communication abilities of the child is that compared with other measured child development domains, communication abilities are developed during interactions, and the most frequent person a child interacts with is the mother. Thus, poor communication abilities of the child are a result of the combined lack of mother–child interactions and MDS which have a significant interactive effect. Because moderating effects of mother–child interactions have not been routinely examined in the literature, additional studies are needed to confirm our findings.

Although the current study improves a broader understanding of the role of mother–child interactions in the associations between MDS and child development outcomes, several limitations should be considered. First, as the analyses were carried out using cross-sectional data, no causal interpretations can be made. Our study design did not allow us to determine whether MDS caused poor mother–child interactions and poor child development rather than the reverse or a more cyclic causal relationship. Our study design also limited our ability to test the mediating effects, so we must be careful not to overstate conclusions about mediation from cross-sectional analyses. Future longitudinal data with more appropriate statistical methods, such as latent variable models, is needed for further verification. Second, this data was obtained through maternal self-reporting. It’s inevitable for information bias to occur because of overreporting and underreporting. MDS may influence how a mother perceives her child development outcomes, which could confound the association (Foster, Garber & Durlak, 2008). Third, our study used secondary data not specifically designed for our research objectives. Although we adjusted for potential confounding covariates, we cannot rule out the possibility of residual confounding because there are a significant number of factors that influence early childhood development. Besides, the covariates we mainly adjusted were from the characteristics of the child rather than of the mother. Some critical factors of the mother, such as marital satisfaction, social support and other mental disorders, were not available for our study.

## Conclusions

The current study confirms the negative associations between MDS and child communication, gross motor function, fine motor function, problem solving and personal social abilities. Mother–child interactions partly mediated these associations. Furthermore, mother–child interactions moderated the negative associations between MDS and communication abilities of the child. Due to the cross-sectional design of this study, these associations require further investigation in prospective studies.

##  Supplemental Information

10.7717/peerj.11060/supp-1 Supplemental Information 1 Raw data

## Additional Information and Declarations

### Data Availability

The following information was supplied regarding data availability: Raw measurements are available as a Supplementary File.
